# Supplementary material for: Unveiling the Bioactive Potential of the Invasive Jellyfish Phyllorhiza punctata Through Integrative Transcriptomic and Proteomic Analyses
Source: Biomolecules. 2025 Aug 4;15(8):1121. doi: 10.3390/biom15081121 (PMC12383608; doi:10.3390/biom15081121)
Supplement: Supplementary file 1 [file biomolecules-15-01121-s001.zip › Suplementary Material/FileS3/PEPFOLD4/XP_065064552_11665/clusters.html]

 PEP-FOLD clusters


Download archive of all models  here   

|  |  |  |
| --- | --- | --- |
| # | file | sOPEP |
| # cluster 1 - Size: 1 |  |  |
|  | XP\_065064552\_11665-model1 | -3.50732 |
| # cluster 2 - Size: 2 |  |  |
|  | XP\_065064552\_11665-model2 | -3.23851 |
|  | XP\_065064552\_11665-model2.1 | -3.19928 |
| # cluster 3 - Size: 2 |  |  |
|  | XP\_065064552\_11665-model3 | -3.20176 |
|  | XP\_065064552\_11665-model3.1 | -3.12545 |
| # cluster 4 - Size: 3 |  |  |
|  | XP\_065064552\_11665-model4 | -3.17884 |
|  | XP\_065064552\_11665-model4.1 | -3.15417 |
|  | XP\_065064552\_11665-model4.2 | -3.04181 |
| # cluster 5 - Size: 2 |  |  |
|  | XP\_065064552\_11665-model5 | -3.1629 |
|  | XP\_065064552\_11665-model5.1 | -3.10097 |
| # cluster 6 - Size: 20 |  |  |
|  | XP\_065064552\_11665-model6 | -3.13354 |
|  | XP\_065064552\_11665-model6.1 | -3.09941 |
|  | XP\_065064552\_11665-model6.2 | -3.0956 |
|  | XP\_065064552\_11665-model6.3 | -3.08702 |
|  | XP\_065064552\_11665-model6.4 | -3.08274 |
|  | XP\_065064552\_11665-model6.5 | -3.05813 |
|  | XP\_065064552\_11665-model6.6 | -3.05147 |
|  | XP\_065064552\_11665-model6.7 | -3.0328 |
|  | XP\_065064552\_11665-model6.8 | -3.02596 |
|  | XP\_065064552\_11665-model6.9 | -3.02468 |
|  | XP\_065064552\_11665-model6.10 | -3.01608 |
|  | XP\_065064552\_11665-model6.11 | -3.00567 |
|  | XP\_065064552\_11665-model6.12 | -2.99369 |
|  | XP\_065064552\_11665-model6.13 | -2.99159 |
|  | XP\_065064552\_11665-model6.14 | -2.98799 |
|  | XP\_065064552\_11665-model6.15 | -2.98611 |
|  | XP\_065064552\_11665-model6.16 | -2.98221 |
|  | XP\_065064552\_11665-model6.17 | -2.97673 |
|  | XP\_065064552\_11665-model6.18 | -2.96943 |
|  | XP\_065064552\_11665-model6.19 | -2.93757 |
| # cluster 7 - Size: 3 |  |  |
|  | XP\_065064552\_11665-model7 | -3.10866 |
|  | XP\_065064552\_11665-model7.1 | -3.01905 |
|  | XP\_065064552\_11665-model7.2 | -2.99963 |
| # cluster 8 - Size: 4 |  |  |
|  | XP\_065064552\_11665-model8 | -3.10638 |
|  | XP\_065064552\_11665-model8.1 | -3.05237 |
|  | XP\_065064552\_11665-model8.2 | -2.97489 |
|  | XP\_065064552\_11665-model8.3 | -2.96208 |
| # cluster 9 - Size: 2 |  |  |
|  | XP\_065064552\_11665-model9 | -3.08607 |
|  | XP\_065064552\_11665-model9.1 | -2.96398 |
| # cluster 10 - Size: 3 |  |  |
|  | XP\_065064552\_11665-model10 | -3.08428 |
|  | XP\_065064552\_11665-model10.1 | -3.00476 |
|  | XP\_065064552\_11665-model10.2 | -2.94589 |
| # cluster 11 - Size: 2 |  |  |
|  | XP\_065064552\_11665-model11 | -3.08305 |
|  | XP\_065064552\_11665-model11.1 | -2.98052 |
| # cluster 12 - Size: 2 |  |  |
|  | XP\_065064552\_11665-model12 | -3.07667 |
|  | XP\_065064552\_11665-model12.1 | -2.98967 |
| # cluster 13 - Size: 3 |  |  |
|  | XP\_065064552\_11665-model13 | -3.07261 |
|  | XP\_065064552\_11665-model13.1 | -3.05742 |
|  | XP\_065064552\_11665-model13.2 | -2.9576 |
| # cluster 14 - Size: 1 |  |  |
|  | XP\_065064552\_11665-model14 | -3.06993 |
| # cluster 15 - Size: 3 |  |  |
|  | XP\_065064552\_11665-model15 | -3.05576 |
|  | XP\_065064552\_11665-model15.1 | -3.02513 |
|  | XP\_065064552\_11665-model15.2 | -2.96189 |
| # cluster 16 - Size: 1 |  |  |
|  | XP\_065064552\_11665-model16 | -3.05415 |
| # cluster 17 - Size: 5 |  |  |
|  | XP\_065064552\_11665-model17 | -3.04738 |
|  | XP\_065064552\_11665-model17.1 | -3.04151 |
|  | XP\_065064552\_11665-model17.2 | -3.00808 |
|  | XP\_065064552\_11665-model17.3 | -2.99286 |
|  | XP\_065064552\_11665-model17.4 | -2.97623 |
| # cluster 18 - Size: 4 |  |  |
|  | XP\_065064552\_11665-model18 | -3.02554 |
|  | XP\_065064552\_11665-model18.1 | -3.00706 |
|  | XP\_065064552\_11665-model18.2 | -2.95789 |
|  | XP\_065064552\_11665-model18.3 | -2.91882 |
| # cluster 19 - Size: 1 |  |  |
|  | XP\_065064552\_11665-model19 | -3.02253 |
| # cluster 20 - Size: 3 |  |  |
|  | XP\_065064552\_11665-model20 | -3.02081 |
|  | XP\_065064552\_11665-model20.1 | -2.94372 |
|  | XP\_065064552\_11665-model20.2 | -2.9116 |
| # cluster 21 - Size: 1 |  |  |
|  | XP\_065064552\_11665-model21 | -3.0159 |
| # cluster 22 - Size: 1 |  |  |
|  | XP\_065064552\_11665-model22 | -3.00928 |
| # cluster 23 - Size: 1 |  |  |
|  | XP\_065064552\_11665-model23 | -3.00897 |
| # cluster 24 - Size: 1 |  |  |
|  | XP\_065064552\_11665-model24 | -3.00194 |
| # cluster 25 - Size: 1 |  |  |
|  | XP\_065064552\_11665-model25 | -2.99799 |
| # cluster 26 - Size: 1 |  |  |
|  | XP\_065064552\_11665-model26 | -2.99156 |
| # cluster 27 - Size: 3 |  |  |
|  | XP\_065064552\_11665-model27 | -2.99071 |
|  | XP\_065064552\_11665-model27.1 | -2.95837 |
|  | XP\_065064552\_11665-model27.2 | -2.93404 |
| # cluster 28 - Size: 1 |  |  |
|  | XP\_065064552\_11665-model28 | -2.98944 |
| # cluster 29 - Size: 4 |  |  |
|  | XP\_065064552\_11665-model29 | -2.98707 |
|  | XP\_065064552\_11665-model29.1 | -2.97463 |
|  | XP\_065064552\_11665-model29.2 | -2.97463 |
|  | XP\_065064552\_11665-model29.3 | -2.96293 |
| # cluster 30 - Size: 1 |  |  |
|  | XP\_065064552\_11665-model30 | -2.98063 |
| # cluster 31 - Size: 1 |  |  |
|  | XP\_065064552\_11665-model31 | -2.97444 |
| # cluster 32 - Size: 2 |  |  |
|  | XP\_065064552\_11665-model32 | -2.9704 |
|  | XP\_065064552\_11665-model32.1 | -2.94455 |
| # cluster 33 - Size: 1 |  |  |
|  | XP\_065064552\_11665-model33 | -2.95121 |
| # cluster 34 - Size: 1 |  |  |
|  | XP\_065064552\_11665-model34 | -2.95071 |
| # cluster 35 - Size: 2 |  |  |
|  | XP\_065064552\_11665-model35 | -2.94911 |
|  | XP\_065064552\_11665-model35.1 | -2.92807 |
| # cluster 36 - Size: 1 |  |  |
|  | XP\_065064552\_11665-model36 | -2.94806 |
| # cluster 37 - Size: 2 |  |  |
|  | XP\_065064552\_11665-model37 | -2.94624 |
|  | XP\_065064552\_11665-model37.1 | -2.91652 |
| # cluster 38 - Size: 1 |  |  |
|  | XP\_065064552\_11665-model38 | -2.94573 |
| # cluster 39 - Size: 2 |  |  |
|  | XP\_065064552\_11665-model39 | -2.93803 |
|  | XP\_065064552\_11665-model39.1 | -2.9366 |
| # cluster 40 - Size: 1 |  |  |
|  | XP\_065064552\_11665-model40 | -2.93634 |
| # cluster 41 - Size: 1 |  |  |
|  | XP\_065064552\_11665-model41 | -2.93132 |
| # cluster 42 - Size: 1 |  |  |
|  | XP\_065064552\_11665-model42 | -2.92983 |
| # cluster 43 - Size: 1 |  |  |
|  | XP\_065064552\_11665-model43 | -2.90629 |
| # cluster 44 - Size: 1 |  |  |
|  | XP\_065064552\_11665-model44 | -2.90353 |
|  |  |  |
